# A core-shell-shell nanoplatform upconverting near-infrared light at 808 nm for luminescence imaging and photodynamic therapy of cancer

**DOI:** 10.1038/srep10785

**Published:** 2015-06-02

**Authors:** Fujin Ai, Qiang Ju, Xiaoman Zhang, Xian Chen, Feng Wang, Guangyu Zhu

**Affiliations:** 1 Department of Biology and Chemistry, City University of Hong Kong, Kowloon Tong, Hong Kong SAR; 2 Department of Physics and Materials Science, City University of Hong Kong, Kowloon Tong, Hong Kong SAR; 3 City University of Hong Kong Shenzhen Research Institute, Shenzhen, P. R. China

## Abstract

Upconversion nanoparticles (UCNPs) have been extensively explored for photodynamic therapy (PDT) and imaging due to their representative large anti-Stokes shifts, deep penetration into biological tissues, narrow emission bands, and high spatial-temporal resolution. Conventional UCNP-based PDT system, however, utilizes exitation at 980 nm, at which water has significant absorption, leading to a huge concern that the cell killing effect is from the irradiation due to overheating effect. Here we report an efficient nanoplatform using 808-nm excited NaYbF_4_:Nd@NaGdF_4_:Yb/Er@NaGdF_4_ core−shell−shell nanoparticles loaded with Chlorin e6 and folic acid for simultaneous imaging and PDT. At this wavelength, the absorption of water is minimized. High energy transfer efficiency is achieved to generate cytotoxic singlet oxygen. Our nanoplatform effectively kills cancer cells in concentration-, time-, and receptor-dependent manners. More importantly, our nanoplatform is still able to efficiently generate singlet oxygen beneath 15-mm thickness of muscle tissue but 980 nm excitation cannot, showing that a higher penetration depth is achieved by our system. These results imply that our nanoplatform has the ability to effectively kill intrinsic tumor or the center of large tumors through PDT, which significantly improves the anticancer efficacy using UCNP-based PDT system and broadens the types of tumors that could be cured.

Photodynamic therapy (PDT) has a long history to treat cancer patients and has now been widely used in the clinic against various types of cancer^1^,^2^. PDT utilizes tissue oxygen and photosensitizers that are excited by visible light to generate highly cytotoxic singlet oxygen (^1^O_2_) and other reactive oxygen species (ROS), which damage cancer cells and lead to cell death^2^. Compared with conventional chemotherapy, PDT is able to specifically eradicate tumors by controlling the location of light exposure^3^. The activation wavelength of most clinically used photosensitizers, however, is usually in a spectrum window of around 630-700 nm^1^, in which tissue penetration depth is limited. Thus, PDT has limited therapeutic effect against internal or large tumors^4^. In addition, the biodistribution of photosensitizers is not controlled, resulting in toxicity issues^3^,^5^.

One way to avoid the aforementioned issues is to shift the excitation wavelength to near infrared (NIR) area. In this region, biological tissues have the minimal light absorption, and increased penetration depth could be achieved in the tumor site^4^. Upconversion nanoparticles (UCNPs) excited by NIR light greatly meet the demands^6^,^7^,^8^. Lanthanide-doped UCNPs have shown exciting biomedical applications including bioimaging, sensing, and drug delivery^9^,^10^,^11^,^12^,^13^,^14^,^15^,^16^,^17^,^18^,^19^,^20^,^21^,^22^,^23^,^24^,^25^,^26^,^27^. In the upconversion process, two or more low-energy photons from NIR light are absorbed to produce higher energy emission in the visible region, which could be further applied in a PDT process^28^,^29^,^30^,^31^,^32^,^33^. The utilization of NIR light rather than visible light as the excitation source ensures low photo-damage, low-autofluorescence background, and deep penetration into biological tissue, which, in combination with the long lifetime of the lanthanide-doped UCNPs, leads to high spatial-temporal resolution^34^. Conventional UCNPs use Yb^3+^ as the light harvesting ion and can respond to a narrow NIR band at around 980 nm^35^,^36^. Nanoplatforms utilizing such UCNPs and different photosensitizers for PDT of cancer have been extensively explored^37^,^38^,^39^. The absorption of water at 980 nm, however, is significant^40^,^41^, leading to an overheating issue and relatively low penetration depth of tissue. This drawback has significantly limited the biomedical applications of conventional UCNPs for imaging and photodynamic therapy.

Efforts have been made to adjust the excitation window of UCNPs. Different metal ions and organic dyes have been incorporated into the system as sensitizers to upconvert NIR light in the medical spectral window (i.e. ~700-900 nm)^36^. At around 800 nm, water has minimized absorption, and there is limited overheating effect. Very recently, Nd^3+^-sensitized upconversion process utilizing a new excitation wavelength at around 800 nm has been reported^35^,^36^,^40^,^41^,^42^,^43^. In the core-shell nanoparticles, the successive Nd^3+^→Yb^3+^→ activator energy transfer enables the excitation at a shorter wavelength of 808 nm^41^. Another report showed that Nd^3+^-sensitized core-shell nanoparticles containing a series of lanthanide activators have remarkably enhanced upconversion luminescence, which provide a more sensitive biomarker for bioimaging without autofluorescence^35^. Most of the reports on Nd^3+^-sensitized UCNPs using 808 nm excitation focused on the fabrication of nanoparticles, and the biomedical application of UCNPs excited by 808 nm, especially on PDT, is still a nascent area.

Herein we fabricated a nanoplatform based on NaYbF_4_:Nd@NaGdF_4_:Yb/Er@NaGdF_4_ core−shell−shell nanoparticles that convert NIR light for simultaneous fluorescence imaging and photodynamic therapy against cancer. This highly efficient system utilizes an energy transfer from 808 nm NIR light to two upconversion luminescence bands at around 550 and 660 nm for simultaneous imaging and therapy (Fig. 1). Chlorin e6 (Ce6), a commonly used and highly efficient photosensitizer, was covalently conjugated with surface-functionalized core-shell-shell nanoparticles at a high efficiency of around 4,000 molecules per nanoparticle^44^. The energy transfer from nanoparticles to photosensitizers was confirmed by upconversion luminescence spectra, luminescence decay lifetimes, and their ability to generate singlet oxygen. The biomedical applications of our nanoplatform were further illustrated by *in vitro* fluorescence imaging using different cancer cells and by its killing cancer cells in a very effective way upon 808 nm irradiation for just 5 min. To the best of our knowledge, the present study is the first to show the biomedical applications of a nanoplatform utilizing NaYbF_4_:Nd@NaGdF_4_:Yb/Er@NaGdF_4_ core−shell−shell nanoparticles that convert NIR light at 808 nm for its biocompatibility, imaging, and PDT ability. Our study paves the way for the further development of such nanoparticle-based theranostic agents with high energy transfer efficiency and minimized overheating effects.

## Results

**Figure 2:**
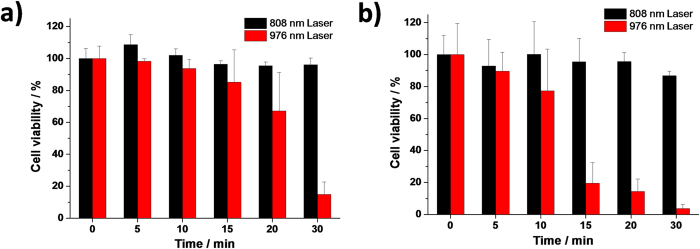
Cell viability of **a**) KB cells and **b**) A549 cells along under different time of 808 nm and 976 nm laser irradiation (1 W/cm^2^).

In the clinical PDT, tumor site will be irradiated by light for a great amount of time. A prerequisite of an ideal nanoplatform upconverting NIR light into visible light for PDT is that the normal tissues shielding the tumor site along the irradiation pathway will not be damaged by the light source. We therefore tested the viability of human cells upon irradiation by 808 nm NIR (1 W/cm^2^). KB, a folate receptor (FR)-expressing human mouth epidermal carcinoma cell line, and A549, a FR-negative human non-small cell lung cancer cell line, were irradiated for up to 30 min and further incubated for 48 h before cell viability measurements. Irradiation at 976 nm (1 W/cm^2^) was used as a control (Fig. 2). Cell viability upon 808 nm laser irradiation did not change significantly but it greatly reduced upon 976 nm laser irradiation. For example, after 30 min irradiation, 96.1% of KB cells remained confluent when subjected to an 808 nm laser but the cell viability dramatically reduced to 15.0% when a 976 nm laser was used. The identical effect was observed in A549 cells. This result indicates that the 808 nm laser irradiation itself does not affect the cell viability for biomedical studies and therefore does not contribute to the cell killing effect from a nanoplatform upconverting 808 nm NIR for PDT. The effect from a 976 nm laser itself on the cell viability, however, especially when the irradiation is longer than 10 min, is a huge concern. One of the best explanations for this phenomenon is the overheating effect from a 976 nm laser, which has been observed previously^35^,^40^,^41^. We also measured the viability of A549 and KB cells upon irradiation at 808 nm at different power densities ranging from 0 to 6 W/cm^2^ and at different time points at 6 W/cm^2^ (see Supplementary Fig. S1 online). The results clearly show that the irradiation at 808 nm has negligible effect on the cell growth of both A549 and KB cells.

**Figure 3:**
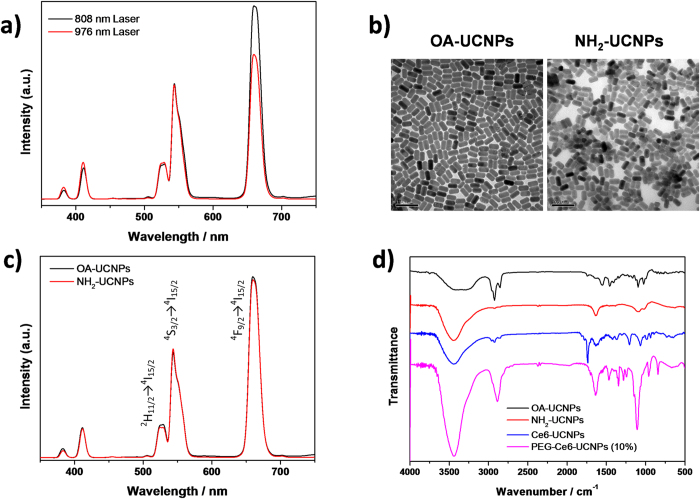
**a**) Photoluminescent spectra of UCNPs under 808 nm and 976 nm excitation (6 W/cm^2^). **b**) TEM images of OA-UCNPs (left) and NH_2_-UCNPs (right). **c**) Photoluminescent spectra of OA-UCNPs and NH_2_-UCNPs under 808 nm laser irradiation (6 W/cm^2^). **d**) FT-IR spectra of OA-UCNPs, NH_2_-UCNPs, Ce6-UCNPs, and PEG-Ce6-UCNPs.

We next constructed a biocompatible and tumor-targeting nanoplatform converting 808 nm NIR light to singlet oxygen for PDT. A NaYbF_4_:Nd@NaGdF_4_:Yb/Er@NaGdF_4_ core−shell−shell nanostructure was synthesized to carry out the photon upconversion^36^. In comparison with Nd^3+^-sensitized upconversion nanoparticles developed by the groups of Yan and Liu^35^,^41^, our nanostructure accommodates a higher content of Yb^3+^, which promotes the red emission band of Er^3+^ to ensure efficient energy transfer to Ce6 displaying an absorption maximum at ~660 nm. It is noted that the nanostructure also upconverts 976 nm NIR light due to the NaGdF_4_:Yb/Er inner shell layer (Fig. 3a), thereby providing a continent platform for assessing the effect of irradiation wavelength on the PDT.

The nanoparticles were functionalized with photosensitizers and cancer-targeting moieties. Amino-functionalized UCNPs (NH_2_-UCNPs) capped with 2-aminoethyl dihydrogen phosphate (AEP) were fabricated by a phase transfer process from hydrophobic oleic acid-UCNPs (OA-UCNPs). As confirmed by TEM, NH_2_-UCNPs showed the same morphology and high monodispersity compared with OA-UCNPs (Fig. 3b). The upconversion luminescence spectrum of NH_2_-UCNPs remained the same as that of OA-UCNPs (Fig. 3c), showing that the surface modification with amino groups hardly alters the optical properties of the nanoparticles. Two emission bands at 520-540 nm and 540-560 nm in the green spectral region are attributed to Er transitions from ^2^H_11/2_ to ^4^I_15/2_ and ^4^S_3/2_ to ^4^I_15/2_, respectively, and the emission band in the red spectral region at 640-680 nm is due to the Er transition from ^4^F_9/2_ to ^4^I_15/2_ (Fig. 3c). FT-IR analysis showed that the peaks belonging to C-H stretching in oleic acid at 2920 and 2850 cm^−1^ in OA-UCNPs disappeared after phase transfer (Fig. 3d). The peaks at 1633 and 1383 cm^−1^ in NH_2_-UCNPs were from N-H bending and C-N stretching vibration, respectively, confirming the successful surface modification with amino groups. Different amount of Ce6 was covalently loaded onto NH_2_-UCNPs via a carbodiimide cross-linking reaction between the amino groups on UCNPs and the carboxylate groups of Ce6^45^. FT-IR analysis of the Ce6-loaded UCNPs showed that the peak at 1637 cm^−1^ was associated with the C=O stretching vibration from the amide group and the 1736 cm^−1^ peak was from the unreacted carboxylate groups in Ce6 since the molecule contains three carboxylate groups (Fig. 3d). Finally, the nanoparticles were capped with PEG and folic acid (FA)-PEG to obtain FA-PEG-Ce6-UCNPs (Scheme 1). The functionalization of UCNPs with Ce6, PEG, and FA was further confirmed by FT-IR and UV-Vis spectroscopy (Fig. 3d, Supplementary Figs. S2 and S3). It is estimated that 1,000 FA molecules were conjugated on each nanoparticle^46^.

**Figure 4:**
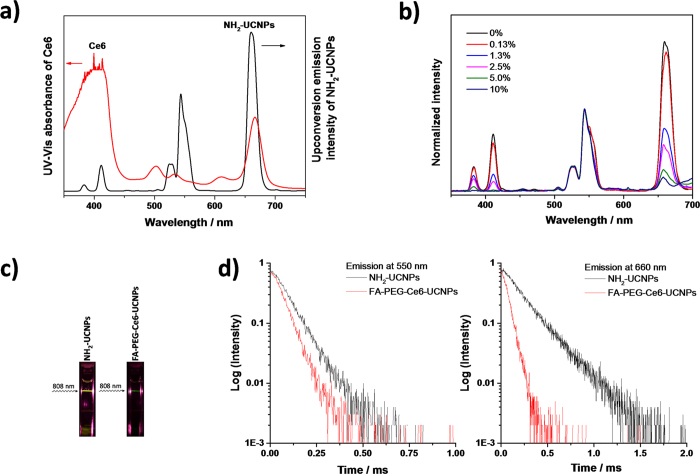
**a**) Photoluminescent spectrum of NH_2_-UCNPs under 808 nm laser (6 W/cm^2^) irradiation (black) and UV-Vis spectrum of Chlorin e6 (red). **b**) Normalized photoluminescent spectra (by the emission peak at 550 nm) of PEG-Ce6-UCNPs with different loading amount under 808 nm laser irradiation. **c**) Photos of NH_2_-UCNPs and FA-PEG-Ce6-UCNPs under 808  nm laser (6 W/cm^2^) irradiation. **d**) Luminescence decay curves of the emission at 550 nm and 660 nm of NH_2_-UCNPs and FA-PEG-Ce6-UCNPs excited by an 808 nm flash laser.

The energy transfer process from the nanoparticles to the photosensitizers (Ce6) was first characterized by photoluminescence spectroscopy. We measured the UV-vis absorbance of Ce6 and confirmed that the absorption peak matches well with the emission peak of NH_2_-UCNPs at around 660 nm (Fig. 4a). Steady-state upconversion luminescence measurements showed that increasing amount of loaded Ce6 resulted in decreasing emission at around 660 nm but not at around 550 nm from Ce6-loaded nanoparticles, indicating the selective energy transfer to Ce6 (Fig. 4b). The Förster resonance energy transfer (FRET) efficiency was determined to be over 70% according to the emission intensity of UCNPs at 660 nm with and without Ce6 modification^31^. This selective energy transfer can be visualized by NIR excitation of the NH_2_-UCNPs and the Ce6-loaded UCNPs. A clear color change from the yellow color of the NH_2_-UCNPs to the green color of the FA-PEG-Ce6-UCNPs upon 808 nm excitation was recorded by a regular digital camera (Fig. 4c). The energy transfer process was further studied by measuring the temporal behavior of upconversion luminescence. The emission decay curves of the NH_2_-UCNPs and the FA-PEG-Ce6-UCNPs at 550 and 660 nm are shown in Fig. 4d. In the presence of Ce6, the average decay time at 550 nm slightly decreased from 84.16 μs to 64.03 μs, partly attributing to the weak absorption of Ce6 at 550 nm (Fig. 4a). Notably, the decay time at 660 nm significantly decreased from 218.92 μs to 63.61 μs. This effect was attributed to the strong absorption of Ce6 at around 660 nm, confirming the selective and highly efficient energy transfer from the UCNPs to the photosensitizers.

**Figure 5:**
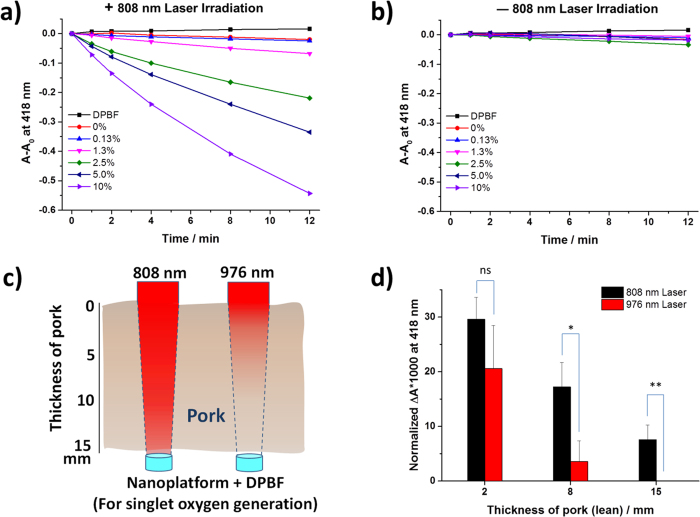
**a**) Singlet oxygen generation ability under 808 nm laser irradiation (3 W/cm^2^) and **b**) without 808 nm laser irradiation by DPBF assay with different Ce6 loading amount. **c**) Scheme of the singlet oxygen test in the presence of pork muscle tissue placed between 808 nm or 976 nm laser (6  W/cm^2^) and the UCNPs/DPBF solution. **d**) Singlet oxygen generation in the presence of different thickness of pork muscle tissue under 808 nm or 976 nm laser (6 W/cm^2^). ns, not significant; *, p < 0.05 ; **, p < 0.01.

The ability of PEG-Ce6-UCNPs to generate cytotoxic ^1^O_2_ was assessed using one of the most well-known chemical probes, 1,3-diphenylisobenzofuran (DPBF)^28^. DPBF reacts with ^1^O_2_ rapidly and specifically with high sensitivity and is inert with the ground state (triplet) molecular oxygen nor with the superoxide anion. *o*-Dibenzoylbenzene was formed through a [4 + 2] cycloaddition of ^1^O_2_ after oxidation, inducing the bleaching of DPBF, which can be measured spectroscopically^47^. Under 808 nm laser irradiation at a power density of 3 W/cm^2^, UCNPs without Ce6 loading were unable to induce the bleaching of DPBF, indicating that UCNPs themselves cannot generate ^1^O_2_ under an 808 nm laser. Notably, UCNPs loaded with various amounts of Ce6 induced significant DPBF bleaching, especially at a higher loading amount, confirming the efficient generation of ^1^O_2_ by our UCNP-based nanoplatform (Fig. 5a). Without NIR laser irradiation, the bleaching of DPBF in the presence of different Ce6 loaded samples was negligible (Fig. 5b). Since 10% (w/w) of Ce6 loading achieved the highest ^1^O_2_ generation ability, we used this sample for the following biological tests.

A deeper tissue penetration depth is pivotal for broader applications of PDT. To compare the singlet oxygen generation ability of our nanoplatform beneath different depths of tissue, pork muscle tissues of varying thickness were located between the NIR power source and the PEG-Ce6-UCNPs samples, and the singlet oxygen generation upon 5 min irradiation at 808 nm or 976 nm with the same intensity was measured using DPBF assay (Fig. 5c). The decrease of absorbance at 418 nm in the presence of pork muscle tissue was normalized to that without pork muscle tissue for 808 nm and 976 nm irradiation, respectively (Fig. 5d). Singlet oxygen generation ability of the nanoparticle system decreased upon different thickness of pork under both 808 nm and 976 nm excitation wavelengths but the ability decreased more quickly for 976 nm. For instance, when a pork muscle tissue of 8 mm thickness was applied, the generated amount of singlet oxygen decreased to 17.2% and 3.6% of the corresponding controls for 808 nm and 976 nm excitation, respectively. For 15 mm thickness, the singlet oxygen generation remains at 7.6% for 808 nm excitation but there was no detectable singlet oxygen generation for 976 nm excitation. In another word, in the potential clinical application, when our nanoplatform is under 15 mm thickness of the patient tissue and excited by a NIR light source, 808 nm excitation is still able to generate singlet oxygen, although the amount will decrease, but 976 nm excitation cannot. Therefore, 808 nm excitation has deeper tissue penetration depth than 976 nm excitation to generate singlet oxygen using our nanoplatform. Together with our results from the cell viability test using different NIR light sources (Fig. 2), our nanoplatform is able to be effective even deep in the tissue but will be safe using 808 nm excitation. Therefore, compared with the system using excitation at 976 nm, our system is more applicable for an efficient PDT.

To ensure the energy transfer process within the cells, Ce6 should be stably conjugated on the surface of the nanoparticles and should not release quickly from the complex. We therefore measured the release profile of Ce6 in UCNPs loaded with the photosensitizers. The release of free Ce6 was monitored by dialysis of FA-PEG-Ce6-UCNPs in PBS of pH 7.4 and pH 6.0. The result is shown in Supplementary Figure S4. At pH 7.4 after 2 h, 95.3% of Ce6 still remained in the nanoparticles and the number was 90.3% after 4 h, indicating that most of the photosensitizers stayed in the nanoparticles. The identical stability was observed at pH 6.0, which is the pH of early endosome^48^. Other reports have supported the notion that most of the nanoparticles have entered cells within this time frame^30^,^46^. Therefore, our nanoconstruct is stable and will still have high energy transfer efficiency after their entrance into cells.

**Figure 6:**
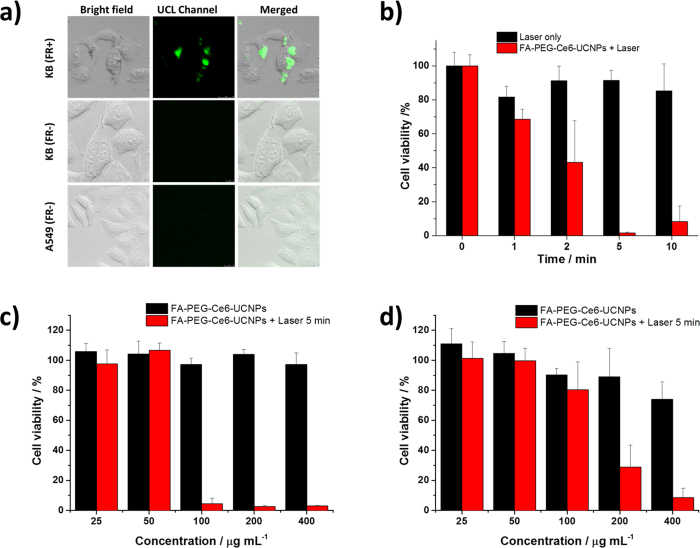
**a**) Upconversion luminescence imaging (λ_em_ = 500-700 nm) of KB (FR+), KB (FR−) and A549 cells incubated with 100 μg/mL nanoparticles for 4 h at 37 °C. Overlay of luminescence image and bright field image were also shown. **b**) Cell viability of KB cells treated with 200 μg/mL FA-PEG-Ce6-UCNPs under 808 nm laser irradiation (6 W/cm^2^) for different amount of time. Cell viability of **c**) KB cells and **d**) A549 cells treated by different concentration of FA-PEG-Ce6-UCNPs under 5 min of 808 nm laser irradiation at 6 W/cm^2^.

The bioimaging properties of FA-PEG-Ce6-UCNPs are shown in Fig. 6a. KB, a folate receptor (FR)-expressing human mouth epidermal carcinoma cell line, and A549, a FR-negative human non-small cell lung cancer cell line, were utilized. Cells were treated with 100 μg/mL nanoparticles and washed three times before confocal imaging. Upconversion luminescence of 500-700 nm channel was monitored. Bright field images of both KB and A549 cells treated with nanoparticles showed that the cells maintained normal morphology, indicating the great biocompatibility of the nanoparticles. The nanoparticles were able to enter FR-positive KB cells likely through FR-mediated endocytosis^46^, and the nanoparticles located in the cytoplasm of KB cells. Conversely, in FR-negative A549 cells, few nanoparticles were able to enter cells. To further confirm the specificity of FR-mediated targeting ability, the FR receptor of KB cells was blocked by excessive free FA, assigned as KB (FR-) cells, before incubation with FA-PEG-Ce6-UCNPs. Significantly weak upconversion luminescence signals were observed from this blocked cells compared with KB (FR+) cells (Fig. 6a). These data indicated the selectivity of FA-PEG-Ce6-UCNPs against FR-positive human cancer cells and the capability of our nanoplatform in bioimaging and diagnosis.

NIR-induced *in vitro* photodynamic therapy of cancer cells using our nanoplatform was tested. KB cells were incubated with 200 μg/mL Ce6-loaded nanoparticles before the cells were exposed to 808 nm NIR light at a density of 6 W/cm^2^ for 1, 2, 5, and 10 min. Cell viability was determined using a standard 3-(4,5-dimethylthiazol-2-yl)-2,5-diphenyltetrazolium bromide (MTT) assay. The result is shown in Fig. 6b. When the cells were exposed for 2 min, FA-PEG-Ce6-UCNPs were able to effectively kill cancer cells and 43.2% of the cells remained viable. The cell killing effect was more dramatic when the cells were exposed for a longer time. For example, cell viability reduced significantly to 1.6% and 8.3% under 5 min and 10 min NIR light exposure, respectively, confirming that the nanoplatform was able to kill FR-positive human cancer cells with a very high efficiency. Concentration-dependent cell killing effect was also evaluated. Without exposure to NIR light, viability of KB cells remained above 95% under all the concentrations tested, indicating the dark toxicity of our nanoplatform is negligible, which is the key for its biomedical applications *in vivo*. When the cells were exposed to NIR light for 5 min, the nanoplatform killed KB cells in a concentration-dependent manner (Fig. 6c). For instance, when cells were treated with 100 μg/mL FA-PEG-Ce6-UCNPs, the cell viabilities without laser irradiation and with laser irradiation were 97.2% and 4.5%, respectively, further proving the photodynamic effect of FA-PEG-Ce6-UCNPs. At higher nanoparticle concentrations, KB cells were also effectively killed by the nanoparticles upon irradiation. Conversely, FA-PEG-Ce6-UCNPs were not able to effectively kill A549 cells at 100 μg/mL under the same irradiation condition (Fig. 6d). This result is in agreement with our upconversion luminescence imaging results that under the same concentration the functionalized nanoparticles were hardly able to enter into A549 cells. Notably, at higher concentrations, FA-PEG-Ce6-UCNPs were still able to effectively kill A549 cells upon laser irradiation. This effect was likely from the non-receptor-mediated cellular uptake under high nanoparticle concentrations^46^,^49^.

## Conclusions

In conclusion, we have successfully developed a novel nanoplatform based on NaYbF_4_:Nd@NaGdF_4_:Yb/Er@NaGdF_4_ core−shell−shell nanoparticles excited by 808 nm NIR light to generate ^1^O_2_ via efficient energy transfer from the nanoparticles to photosensitizers. Ce6 was covalently conjugated with surface-functionalized nanoparticles and a high loading efficiency was realized. The energy transfer process was confirmed by upconversion luminescence and luminescence decay. The FA-functionalized nanoparticles were able to efficiently and selectively enter FR-positive human cancer cells. Upon 808 nm irradiation, the Ce6-loaded nanoparticles effectively killed cancer cells in time- and nanoparticle concentration-dependent manners. More importantly, irradiation of 976 nm laser significantly reduced cell viability but the 808 nm laser did not alter cell viability even after an exposure of 30 min. Therefore, our system is able to be exposed to NIR light for a long time without damaging the tissue between the light source and the nanoparticles, leading to safer biomedical applications. In addition, 808 nm NIR has a deeper tissue penetration depth than 976 nm NIR, as evidenced by the observation that our nanoplatform excited by 808 nm NIR was able to generate ^1^O_2_ even under a 15-mm thickness of pork muscle tissue. Thus, compared with conventional UCNP-based PDT system utilizing 976 nm excitation, our nanoplatform bears the ability to effectively kill intrinsic tumor or the center of large tumors through PDT, which significantly improves the anticancer efficacy using UCNP-based PDT system and broadens the types of tumors that could be cured by PDT. Our study paves the way for the further development of this multifunctional core-shell-shell nanoplatform as an effective theranostic agent for diagnosis and PDT of cancer in preclinical and even clinical settings.

## Methods

### Materials

2-aminoethyl dihydrogen phosphate (AEP) was purchased from J&K Scientific Ltd. Chlorin e6 (Ce6) was obtained from Frontier Scientific. H_2_N-PEG-COOH (MW 5,000) was purchased from Nanocs. Methoxy poly(ethylene glycol) succinimidyl ester (mPEG-SC) was from Sinopeg Biotech Co. Ltd. *N*-(3-dimethylaminopropyl)-*N*’-ethylcarbodiimide hydrochloride (EDC), *N*-hydroxysuccinimide (NHS), and 1,3-diphenylisobenzofuran (DPBF) were purchased from Sigma-Aldrich. Folic acid was from Acros Organics. 3-(4,5-dimethylthiazol-2-yl)-2,5-diphenyltetrazolium bromide (MTT) was purchased from Life Technologies. All reagents were used as received. KB cells were kindly provided by Mr. Kenneth K. K. Lau of Department of Biology and Chemistry at the City University of Hong Kong and A549 cells were from American Type Culture Collection (ATCC).

### Preparation of NH_2_-UCNPs

Core-shell-shell upconversion nanoparticles [NaYbF_4_:Nd@NaGdF_4_:Yb/Er@NaGdF_4_] were synthesized following a protocol that we have established previously with modifications^36^. UCNPs capped with hydrophobic oleic acid were transferred to aqueous phase by ligand exchange. Briefly, 30 mg of UCNPs was first precipitated by ethanol and then redispersed in 2 mL of chloroform by sonication. A volume of 15 mL AEP solution containing 100 mg AEP in milli-Q water and ethanol (3:2 v/v) was added to the chloroform solution of UCNPs and the mixture was stirred for 48 h at room temperature. The transfer of UCNPs from the bottom chloroform layer into the upper aqueous layer was confirmed by the emission of aqueous layer under 808 nm laser excitation (data not shown). The aqueous phase was subsequently dialyzed (MWCO, 10K) against deionized water for 1 day at room temperature to remove unreacted AEP. Finally, the sample was lyophilized overnight to obtain NH_2_-UCNPs.

### Modification of NH_2_-UCNPs with chlorin e6

Chlorin e6 was dissolved in dimethyl sulfoxide (DMSO) and excess amount of EDC and NHS was added to activate carboxylate for 30 min at room temperature. NH_2_-UCNPs were resuspended into DMSO and ultrasonicated for 10 min; the solution was then added to the activated Ce6 solution containing trace amount of triethylamine (TEA) and stirred overnight. The solution containing Ce6-UCNPs was dialyzed (MWCO, 10K) against deionized water for 1 day at room temperature followed by lyophilization.

### Modification of H_2_N-PEG-COOH with folic acid

Folic acid was activated by EDC and NHS as mentioned above. H_2_N-PEG-COOH (MW 5,000) was dissolved in DMSO, added into the solution of folic acid containing TEA, and stirred overnight at room temperature. Acetone was added to precipitate the unreacted folic acid. FA-PEG-COOH was dialyzed (MWCO, 10K) against deionized water at room temperature for 2 days followed by lyophilization to obtain a light yellow product.

### Modification of Ce6-UCNPs with PEG

FA-PEG-COOH together with EDC and NHS were first dissolved in DMSO and stirred overnight. Ce6-UCNPs were resuspended in DMSO and sonicated for 5 min. mPEG-SC and activated FA-PEG-COOH in DMSO together with TEA was then added and the mixture was stirred overnight at room temperature. Afterward, the PEG-Ce6-UCNPs were dialyzed (MWCO, 10K) against deionized water and subsequently lyophilized for 1 day.

### Characterizations of modified UCNPs

UV-visible spectrum was recorded on a Shimazu UV-1700 UV-Vis spectrophotometer. Fourier transform infrared (FT-IR) was performed on an AVATAR-360 FT-IR spectrophotometer (Nicolet, USA). Transmission Electron Microscopy (TEM) measurements were carried out on a Philips CM-20 TEM (Philips Technai 12). Photoluminescence (PL) was measured using an F-4600 spectrophotometer (Hitachi) with the excitation source adapted to fiber coupled diode lasers. Decay curves of the emission of at both 550 nm and 660 nm of NH_2_-UCNPs and FA-PEG-Ce6-UCNPs were recorded on FLSP920 fluorescence spectrophotometer (Edinburgh Instruments) equipped with an 808 nm VD-IIA DPSS Laser Driver.

### Detection of singlet oxygen

Singlet oxygen was determined by DPBF according to a previously published report^28^. FA-PEG-Ce6-UCNPs was sonicated and dispersed in DMSO containing 200 μM DPBF. The resulting solution was added in a 96-well plate and irradiated by an 808 nm laser for different amount of time, and the absorbance at 418 nm was recorded. The generation of singlet oxygen by FA-PEG-Ce6-UCNPs bleaches DPBF, thus the decreased absorption of DPBF at 418 nm reflects the singlet oxygen level produced by FA-PEG-Ce6-UCNPs.

### Near infrared (NIR) induced PDT efficiency of modified UCNPs

A549 and KB cells were seeded in 96-well culture plates at a density of 1,500 and 2,000 cells/well, respectively, and cultured in 5% CO_2_ at 37 °C for 48 h. The cells were then treated with different concentrations of FA-PEG-Ce6-UCNPs for 2 h and irradiated with an 808 nm laser for 5 min/well (6 W/cm^2^). The cells were incubated for another 48 h in the dark before the standard MTT assay. Cell viabilities were normalized to the viabilities of untreated cells with irradiation.

### *In vitro* cell imaging

KB (FR+) cells were cultured in FA-free RPMI-1640 medium (Gibco) supplemented with 10% (v/v) fetal bovine serum and 50 units/mL penicillin/streptomycin at 37 °C under humidified air containing 5% CO_2_. For KB (FR-) cells, the KB cells were cultured in RPMI-1640 medium supplemented with free folic acid (1 μg/mL), thus the folate receptors on the KB cells membrane were blocked by free folic acid. A549 cells were cultured in DMEM supplemented with 10% (v/v) fetal bovine serum and 50 units/mL penicillin/streptomycin at 37 °C under humidified air containing 5% CO_2_. Cells were then seeded onto coverglass bottom culture dish and allowed to adhere for 40 h. The cells were incubated in fresh medium containing 100 μg/mL nanoparticles for 4 h and then washed three times with PBS to sufficiently remove excessive nanoparticles. Cell imaging was performed on a Leica SP5 confocal microscope. The upconversion luminescence emission of UCNPs (emission range 500-700 nm) were excited by an 808 nm laser.

## Note

During the preparation of this manuscript, a related work using 808 nm-excited UCNPs for photodynamic therapy was reported (D. Wang, B. Xue, X. Kong, L. Tu, X. Liu, Y. Zhang, Y. Chang, Y. Luo, H. Zhao and H. Zhang, Nanoscale, 2015, 7, 190-197, First published online 03 Nov 2014).

## Additional Information

**How to cite this article**: Ai, F. *et al.* A core-shell-shell nanoplatform upconverting near-infrared light at 808 nm for luminescence imaging and photodynamic therapy of cancer. *Sci. Rep.* **5**, 10785; doi: 10.1038/srep10785 (2015).

## Supplementary Material

Supplementary Information

## Figures and Tables

**Figure 1:**
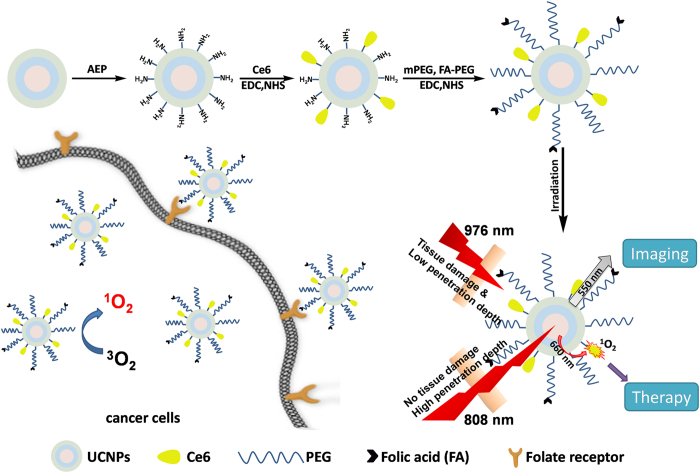
Functionalization of core-shell-shell nanoparticles with photosensitizer Ce6, PEG, and cancer-targeting moiety folic acid (FA) for simultaneous imaging and PDT.
