# Supplementary material for: A core-shell-shell nanoplatform upconverting near-infrared light at 808 nm for luminescence imaging and photodynamic therapy of cancer
Source: Sci Rep. 2015 Jun 2;5:10785. doi: 10.1038/srep10785 (PMC4451683; doi:10.1038/srep10785)
Supplement: Supplementary Information [file srep10785-s1.doc]

SUPPLEMENTARY INFORMATION

A core-shell-shell nanoplatform upconverting near-infrared light at 808 nm for luminescence imaging and photodynamic therapy of cancer

Fujin Ai,^1,3^ Qiang Ju,^2^ Xiaoman Zhang,^1^ Xian Chen,^2^ Feng Wang,^2,3,*^ and Guangyu Zhu^1,3,*^

^1^Department of Biology and Chemistry, City University of Hong Kong, Kowloon Tong, Hong Kong SAR; ^2^Department of Physics and Materials Science, City University of Hong Kong, Kowloon Tong, Hong Kong SAR; ^3^City University of Hong Kong Shenzhen Research Institute, Shenzhen, P. R. China

*Corresponding authors. E-mails: guangzhu@cityu.edu.hk; fwang24@cityu.edu.hk

**Supplementary Figure S1.** Cell viability of A549 and KB cells upon 808 nm laser irradiation a) under diffrent power densities at 0, 1, 3, or 6 W/cm^2^ for 5 min, and b) at different time points (6 W/cm^2^).

**Supplementary Figure S2.** UV-Vis spectra of PEG-Ce6-UCNPs with different loading amount of Ce6.

**Supplementary Figure S3.** UV-Vis spectra of folic acid, FA-PEG-Ce6-UCNPs, PEG-Ce6-UCNPs, and Ce6.

**Supplementary Figure S4.** Release curve of Ce6 in FA-PEG-Ce6-UCNPs in PBS at pH 7.4 and 6.0.
